# Prevalence and Radiographic Patterns of Impacted Third Molars in a Portuguese Population: A Retrospective Orthopantomography (OPG) and Cone-Beam Computed Tomography (CBCT) Study

**DOI:** 10.3390/jcm15031160

**Published:** 2026-02-02

**Authors:** Ana Catarina Pinto, Helena Francisco, Maria Inês Charro, Duarte Marques, Jorge N. R. Martins, João Caramês

**Affiliations:** 1 Faculdade de Medicina Dentária, Universidade de Lisboa, 1600-277 Lisboa, Portugal; mariacharro@edu.ulisboa.pt (M.I.C.); duarte.marques@campus.ul.pt (D.M.); jnrmartins@edu.ulisboa.pt (J.N.R.M.); carames@campus.ul.pt (J.C.); 2 Implantology Institute, 1070-064 Lisbon, Portugal; 3 LIBPhys-FCT UIDB/04559/2020, Faculdade de Medicina Dentária, Universidade de Lisboa, 1600-277 Lisboa, Portugal; 4 Center for Evidence-Based Dental Medicine, Faculdade de Medicina Dentária, Universidade de Lisboa, 1600-277 Lisboa, Portugal

**Keywords:** third molar, impaction, panoramic radiography, orthopantomography, cone-beam computed tomography, inferior alveolar canal, radiographic risk indicators

## Abstract

**Background/Objectives:** Impacted third molars are frequent and may increase surgical complexity, particularly when the mandibular third molar is in close proximity to the inferior alveolar canal (IAC). This study aimed to estimate the prevalence and impaction patterns of third molars in a Portuguese population and to characterize, using a nested CBCT subsample, the three-dimensional relationship between mandibular third molars and the IAC, including cortical integrity and lingual plate thickness. **Methods:** A retrospective observational analysis of 1062 orthopantomograms (OPGs) was performed to determine the prevalence and panoramic patterns using Winter, Pell and Gregory classifications and Rood–Shehab signs. A consecutive CBCT subsample (n = 205) was assessed for IAC position, contact status (no contact; contact with cortical bone; contact without cortical bone), cortical integrity, and lingual plate thickness. Descriptive statistics were complemented by effect sizes to support clinical interpretability. **Results**: The prevalence of impacted third molars was 34.9%, occurring predominantly in the mandible. Vertical angulation was the most prevalent pattern in both jaws. In the CBCT subsample, IAC position and contact patterns varied widely, and loss of cortical integrity was more often observed when panoramic high-risk signs were present. No clinically meaningful left–right asymmetry was identified across key anatomical risk indicators. **Conclusions**: In this Portuguese cohort, impacted third molars showed consistent panoramic patterns, while CBCT provided clinically relevant three-dimensional risk descriptors—particularly IAC contact type and cortical integrity—supporting selective CBCT use based on anatomical risk indicators rather than routine imaging.

## 1. Introduction

Third molar impaction is a frequent condition in oral and maxillofacial practice and remains the subject of ongoing research and debate. Epidemiological studies consistently report mandibular third molars (M3M) as the most frequently impacted teeth, followed by maxillary third molars (Mx3M) and, less commonly, maxillary canines [1,2,3,4,5]. Despite the extensive literature, however, methodological heterogeneity persists, and data at regional and population levels—particularly in Portuguese populations- remain limited.

Recent systematic reviews and meta-analyses report pooled prevalence estimates ranging from approximately 36.9% (95% CI: 33.1–40.7%) per individual to 46.4% (95% CI: 36.7–56.1%) per tooth, with substantial geographic variability [6]. According to this data, the prevalence is higher in Asian countries, with a prevalence of around 24.5% among European populations [6]. A previous study estimated a global prevalence of impacted third molars of 24.4% (95% CI: 18.97–30.80%) among individuals older than 17 years, highlighting how regional differences, methodological variability, and advances in radiographic technology influence reported outcomes [7].

Most studies have shown that impaction predominantly occurs in the mandible rather than in the maxilla, with mesioangular impaction being the most frequent orientation, followed by vertical, distoangular, and horizontal impactions [6,7]. Female sex has been reported as a modest risk factor, while genetic, dietary, and craniofacial factors may also influence impaction patterns [6,8].

Radiographic analysis is critical in the diagnosis and categorization of third molar impaction. The grading system proposed by Pell and Gregory (1933) uses two parameters: 1) the position of the third molar in relation with the ascending ramus of the mandible (Classes I, II and III; and 2) the depth of the impacted third molar, with the occlusal plane of the adjacent second molar as reference (Level A, B or C) [9]. Winter’s (1926) classification categorizes impaction according to the angulation of the third molar in relation to the second molar as mesioangular (the long axis of the impacted third molar is tilted in a mesial direction), distoangular (the long axis of the impacted third molar is tilted distally to the second molar), horizontal, vertical (long axis of both third and second molar are parallel), buccal/lingual obliquity, transverse (horizontally impacted but in a buccal/lingual direction) or inverse (the tooth is reversed) [10].

Due to its two-dimensional representation and susceptibility to image distortion and anatomical superimposition, OPG may provide limited information regarding buccolingual relationships and the exact spatial proximity to adjacent anatomical structures, namely the IAC, when compared with CBCT [11,12,13]. Thus, the combination of OPG and CBCT has significantly improved diagnostic precision, particularly in evaluating the spatial relationship between impacted teeth and critical anatomical structures such as the inferior alveolar nerve and lingual cortical plate [11,12,13,14].

Despite this growing body of evidence, epidemiological data on the prevalence and distribution of third molar impactions in Portuguese populations are scarce and mainly carried out at dental clinics of the undergraduate programs of dental universities, which makes the target population quite specific [15,16,17] and with a population sample of less than 500 individuals [15,18,19]. In addition, these studies traditionally focused only on mandibular third molars and were based on OPG, which is a limitation regarding precision and accuracy.

Evidence-based medicine (EBM), as the basis of modern clinical decision making, emphasizes the use of high-quality evidence from systematic reviews and prevalence studies [20,21]. Prevalence studies provide important epidemiological data that identify population patterns and help to develop prevention and treatment strategies and guidelines [22].

Thus, the primary outcome of the current study is to assess the prevalence of impacted third molars in a Portuguese population, according to demographically related parameters (age and sex) and associated with the side of the jaws (maxilla and mandible) and the degree of impaction (angulation and depth). Secondary outcomes include three-dimensional assessment of mandibular third molars (M3M) in relation to key anatomical structures, specifically: (1) position of the inferior alveolar canal (IAC) relative to the roots of the M3M; (2) linear distance between the M3M roots and the IAC; (3) thickness of the lingual cortical plate.

Accordingly, this investigation was primarily conceived as a large-scale, prevalence-driven epidemiological study based on panoramic radiographs, aiming to characterize the distribution and radiographic patterns of impacted third molars in a Portuguese population. The CBCT analysis should be interpreted strictly as a secondary, nested component of the study, designed to refine the three-dimensional anatomical assessment of selected mandibular third molars with potential surgical relevance. This approach allows the integration of clinically meaningful anatomical information without altering the prevalence-based nature or epidemiological scope of the investigation.

## 2. Materials and Methods

### 2.1. Study and Sample Characteristic

The study design followed the ‘Strengthening the reporting of observational studies in epidemiology’ (STROBE) statement [19] and was previously approved by the Ethics Committee of the Implantology Institute, Lisbon (II2022-02).

A convenience sample of OPG and CBCT images from a pool of Portuguese patients was collected from the database of the Radiology Department of a single health center in Lisbon, Portugal (Implantology Institute, Lisbon). CBCT scans were performed for diverse reasons such as third molars extraction, implants, and root canal treatment. No questionnaire was used; all study outcomes were radiographic and were assessed using established classification systems (Winter, Pell and Gregory; Rood and Shehab) and predefined CBCT evaluation criteria.

### 2.2. Inclusion and Exclusion Criteria

Third molars were classified according to their eruption status as fully erupted, developing, impacted, embedded, or absent. A tooth was considered impacted when it showed complete root development but failed to erupt due to physical obstruction by adjacent structures (teeth, bone, or surrounding tissues) and was not in functional occlusion. Teeth were recorded as fully erupted when in functional occlusion with the opposing teeth, and as developing when only the crown germ was visible radiographically, with evidence of incomplete root formation.

Inclusion criteria included: (1) patients aged 17 years or older, as this age represents the onset of third molar eruption, allowing reliable differentiation between normal eruption and impaction [7,23]; (2) presenting two-thirds or more of root development; (3) and having an OPG scan of adequate diagnostic quality. Eligible patients were consecutively included in the study.

Exclusion criteria included clinical records with incomplete demographic data (missing age or sex), absence or insufficient quality of radiographic exams (blurred, distorted, or with artefacts affecting visualization of the third molar region), and absence of first or second molars in the same quadrant were excluded from further CBCT analysis. Third molars associated with periapical lesions, cystic alterations, or an enlarged pericoronal space exceeding 5 mm were also excluded. Additionally, CBCT scans acquired with an incomplete field of view (FOV) that did not include the full course of the mandibular canal were excluded from analysis.

### 2.3. Image Acquisition and Processing

CBCT scans were acquired using a Planmeca ProMax unit (Planmeca Oy, Helsinki, Finland), in accordance with the manufacturer’s guidelines and following a standardized clinical protocol (80 kV, 15 mA, exposure time 12 s; voxel size 0.20–0.40 mm; field of view 16 × 14 cm; slice thickness 0.20 mm) and analyzed with dedicated three-dimensional imaging software. Voxel size variability was considered during interpretation, given its influence on measurement precision. In the CBCT subsample, 140 scans were acquired with a 0.20 mm voxel size and 65 scans with a 0.40 mm voxel size. In adherence to the ALARA principle philosophy, these image-acquiring parameters were chosen in order to provide an optimal tradeoff between image quality and radiation dosage. It has been shown in earlier studies that voxel dimensions between 0.2 mm and 0.4 mm provide sufficient image detail for visualization of fine anatomical structures such as the inferior alveolar canal (IAC) and the lingual cortical plate without incurring excessively high dosages of radiation. By contrast, smaller voxel dimensions (<0.2 mm) provide minor increments in image detail in terms of marginal augmentation of dosage, and larger voxel dimensions (>0.4 mm) result in reduced spatial and quantitative measurement resolutions [24,25,26,27]. Artifact reduction and noise filtering tools were applied to optimize image quality and enhance diagnostic reliability.

### 2.4. Radiographic Evaluation

Data collection for each patient included demographic data such as age, sex, site of tooth (upper/lower, right/left), and previous extracted teeth. OPG images were used to:

(1) identify the previously defined eruption categories (absent, developing, fully erupted, impacted);
(2) classify third molar angulation and depth according to Winter’s Classification [10], as Mesioangular, Distoangular, horizontal, vertical or other, and Pell & Gregory’s Classification [9], which considers the tooth’s position in relation to the occlusal plane (Positions A, B, or C) and to the mandibular ramus (Classes I, II, or III), respectively;
(3) identify radiographic signs suggestive of proximity to the inferior alveolar canal (IAC) following the diagnostic criteria proposed by Rood and Shehab [28]. These include darkening, narrowing, or deflection of the root, bifid apices, interruption of the cortical outline of the canal, narrowing of the canal, and canal diversion, which have been associated with an increased risk of inferior alveolar nerve (IAN) exposure or injury during third molar extraction [28,29,30].
(4) identify pathologies associated with the third molars such as pericoronitis, caries (in the third or second molars), periodontal pockets, root resorption, and enlarged pericoronal space.

**Figure 1:**
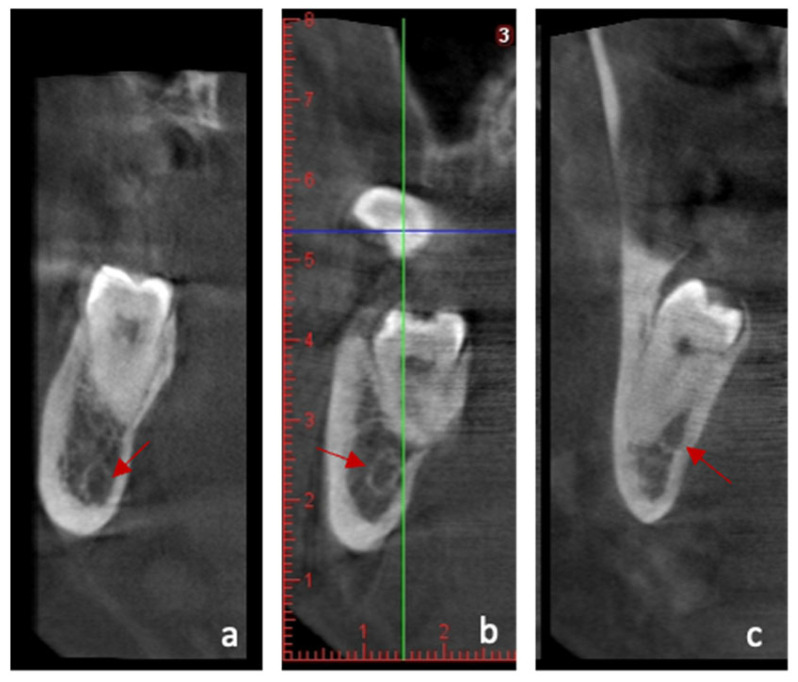
Example of CBCT images that were used to assess the three-dimensional position of M3M and establish possible predictors of surgical risk. For this purpose, three distinct analyses will be performed: position of IAC in relation to the roots of M3M: (**a**) apical; (**b**) buccal; (**c**) lingual. The arrow indicates the position of IAC.

**Figure 2:**
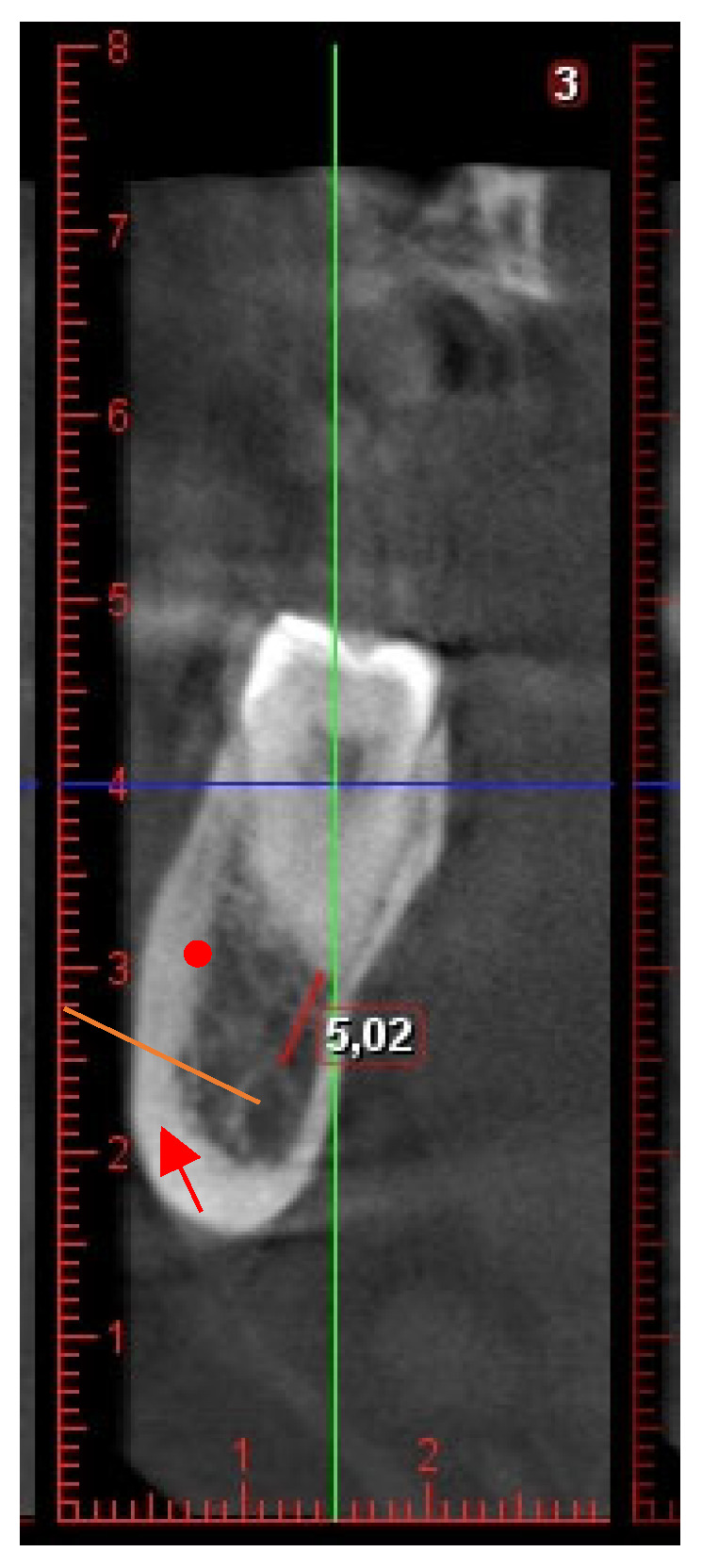
Measurements of the minimum distance from the mandibular third molar root and the tangential line (orange line) to the superior limit of IAC. The IAC is indicated by an arrow, and the closest root surface is indicated by the endpoint of the line used to measure the distance from the superior tangent of the IAC.

**Figure 3:**
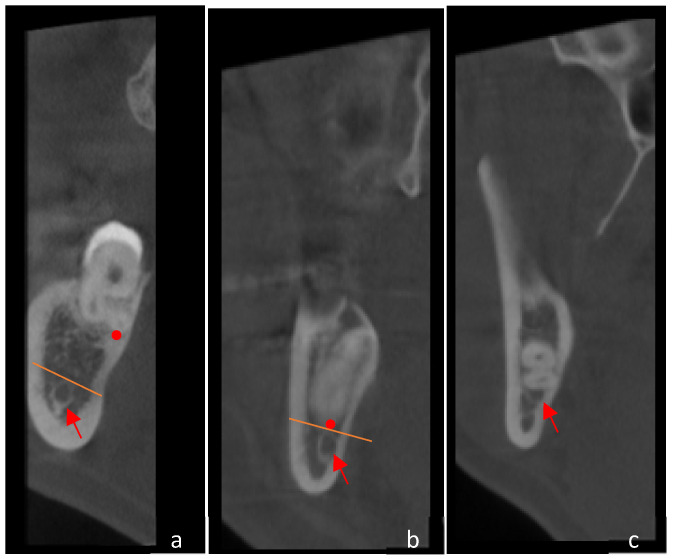
Representative CBCT cross-sectional examples illustrating the three root–IAC relationships used for anatomical risk assessment. (**a**): no IAC contact (a measurable separation is present); (**b**): contact with cortical bone (a cortical boundary remains visible between root and canal); (**c**): contact without cortical bone (loss of cortical integrity), which may indicate a higher likelihood of intimate canal proximity and increased neurosensory risk during surgery. In all images, the IAC is indicated by a red arrow. Additionally, the red sphere represents the root apex, and the orange line indicates the tangent to the superior limit of the IAC. In the last image (**c**), these markers are not shown due to overlap, which would prevent their visualization.

**Figure 4:**
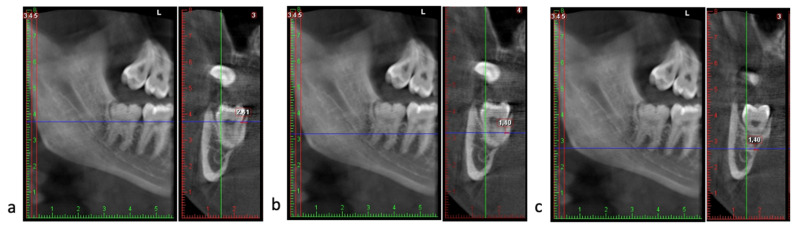
Lingual cortical bone thickness assessment on parasagittal CBCT slices at three levels: (**a**) cementoenamel junction of the second molar; (**b**) mid-root level of the third molar; (**c**) apex of the third molar. Root apices and the lingual cortex are indicated.

CBCT images provided a three-dimensional assessment of the M3M, and four parameters were evaluated for each region of interest:

(1) The position of the inferior alveolar canal (IAC) relative to the roots of the M3M is defined as: apical position, buccal position, lingual position, and interradicular position (Figure 1) [31].

(2) The distance between the M3M roots and the IAC is determined through the evaluation of the smallest distance between the M3M roots and the tangential line to the superior limit of IAC (Figure 2). Unlike previous studies in which contact was defined solely by the absence of a cortical boundary, the present study classified contact whenever the tooth root showed anatomical continuity with the mandibular canal, irrespective of the presence of cortical bone [32,33]. Accordingly, three categories were used to describe the spatial relationship between third molar roots and the mandibular canal: no contact, contact with cortical bone, and contact without cortical bone (Figure 3). This classification was adopted to address situations in which a very thin cortical layer was visible but fell below the voxel-dependent threshold for reliable distance measurement, thereby limiting accurate quantification. This method allowed a more refined assessment of the three-dimensional relationship between the tooth and the IAC [24,25,26,27]. The minimum measurable distance between structures depended on the voxel size of each scan. In scans with a 0.4 mm voxel size, the smallest measurable interval was approximately 1.6 mm, whereas in those with a 0.2 mm voxel size, it was about 0.8 mm. This limitation reflects the dependence of spatial resolution on voxel dimensions, since accurate differentiation requires several consecutive voxels to delineate anatomical boundaries [24,25,26,27].

(3) The thickness of the lingual plate assessed in parasagittal slices at three anatomical levels: (a) at the cementoenamel junction (CEJ) of the mandibular second molar in its most distal portion (closest to the third molar); (b) at mid-root closest to the lingual cortical plate of the third molar, and (c) at the apex of the distal root of the third molar (Figure 4) [34,35].

(4) The relationship between the apex of the third molar root and lingual plate was assessed in parasagittal slices, as they are possible risk factors for lingual nerve (LN) damage, and classified as: (a) type A: presence of bone between the root and the lingual cortex; (b) type B: the root is in contact with the lingual cortex but doesn’t perforate it and (c) the root perforates the lingual cortex [36]. Cases showing lingual cortical perforation or cortical thickness below 1 mm were documented as potential risk indicators for LN injury.
(5) Presence of radiographic signs detected in OPG and the IAC position in relation to the M3M evaluated in CBCT images.

### 2.5. Statistical Analysis

Data were recorded and organized in Microsoft Excel (Microsoft Corp., Redmond, WA, USA) and subsequently analyzed using IBM SPSS Statistics, version 29.0 (IBM Corp., Armonk, NY, USA). Descriptive statistics included absolute and relative frequencies for categorical variables and means with standard deviations (SD) for continuous variables. Associations between categorical variables (e.g., tooth position, eruption status, angulation, radiographic signs of inferior alveolar canal (IAC) proximity) were assessed using the chi-square test of independence. When more than 20% of the expected cell counts were below five, the Monte Carlo simulation method was applied to obtain accurate *p*-values. Standardized adjusted residuals were examined to support post hoc interpretation of statistically significant associations.

For continuous CBCT-derived variables, namely the minimum distance between mandibular third molar roots and the inferior alveolar canal and the thickness of the lingual cortical plate at different anatomical levels, between-group comparisons were performed between mandibular third molars 38 and 48 using independent-samples t-tests. When the assumption of homogeneity of variances was not met, Welch’s correction was applied.

Normality assumptions were assessed using graphical inspection (histograms and Q–Q plots) and Shapiro–Wilk tests. When deviations were observed, findings were confirmed with non-parametric alternatives (Mann–Whitney U), with no change in inference.

In addition to hypothesis testing, effect size measures were systematically calculated to assess the magnitude and potential clinical relevance of observed associations, independently of statistical significance. Cohen’s d was used for continuous variables, and Cramér’s V was calculated for associations between categorical variables. Effect sizes were interpreted according to conventional thresholds (small ≥ 0.2; medium ≥ 0.5; large ≥ 0.8 for Cohen’s d).

For interobserver agreement on radiographic findings, the results were analyzed using Cohen’s Kappa coefficient and interpreted based on criteria proposed by Landis and Koch [37].

Since the nature of this investigation is both descriptive and epidemiological, with a primary focus on the estimation of the prevalence, the use of multivariable modeling was not the main goal. In its place, the use of univariate tests coupled with the estimate of the effect size was considered suitable to characterize associations while preserving interpretability and minimizing the risk of model overfitting.

An a priori sample size estimation was performed to support the adequacy of the available dataset, rather than to define a fixed recruitment target. The estimation was based on the primary outcome, namely the prevalence of impacted third molars assessed on OPG. Considering the prevalence values reported in previous epidemiological studies (30–40%), an expected prevalence of 35% was assumed. Using a 95% confidence level and a maximum absolute precision of approximately ±3.0%, a sample size of approximately 1000 OPG was considered sufficient to provide robust prevalence estimates. Thus, the final OPG dataset (n = 1062 OPGs) was considered adequate for prevalence estimation and subgroup description.

Concerning the CBCT analysis, sample size calculations were also set up with a focus on reaching precision for continuous anatomical variables, rather than to power formal hypothesis testing. Based on conservative variability estimates reported in previous CBCT studies of mandibular third molars, a standard deviation of approximately 2.18 mm was assumed for the root–inferior alveolar canal distance. Targeting a 95% confidence interval half-width of ±0.30 mm, a CBCT sample of approximately 200 mandibular third molars was considered appropriate to provide clinically meaningful precision. Therefore, the available CBCT subsample (n = 205 mandibular third molars) was considered suitable for three-dimensional assessment of canal proximity and lingual cortical plate thickness. All statistical tests were two-tailed, and the level of statistical significance was set at *p* ≤ 0.05.

Given the descriptive and epidemiological nature of this investigation, the primary objective was not to identify independent predictors through multivariable modeling, but rather to characterize prevalence patterns and anatomically relevant radiographic relationships with direct clinical interpretability. The use of univariate statistical analyses complemented by systematic effect size estimation was therefore considered appropriate to minimize model overfitting, reduce collinearity among radiographic classification variables, and preserve transparency in the clinical interpretation of the findings.

## 3. Results

Interobserver agreement: The OPG and CBCT images were analyzed by two independent observers, the leading investigator (C.P.), an experienced oral surgeon, and a second observer, following standardized criteria. Both examiners independently evaluated 20 randomly selected cases, and the interobserver agreement was calculated using Cohen’s Kappa coefficient, which demonstrated an excellent level of agreement (κ = 0.889) according to the interpretation scale proposed by Landis and Koch [37]. In case of disagreement between the two observers, a consensus was reached by discussion.

Sample description: A total of 1548 consecutive patient records were initially screened between January and December 2025. After exclusion of patients younger than 17 years (n = 192), missing clinical records (n = 14), and absence of panoramic radiographs (n = 280), a final sample of 1062 patients was included. These patients corresponded to 1062 orthopantomograms (OPG). Orthopantomography (OPG) is a standardized form of panoramic radiography.

Demographic characteristics: Of the 1062 patients, 619 were female (58.3%), and 443 were male (41.7%). The mean age was 49.47 years (range: 17–94 years). Most patients were older than 36 years (n = 771, 72.6%). Younger age groups were less represented, particularly patients aged 17–20 years (n = 54) and 21–25 years (n = 58).

A total of 397 patients (37.4%) presented absence of all four third molars, while 52 patients (4.9%) showed absence of both mandibular third molars. A total of 1811 third molars were evaluated, of which 1077 were fully erupted, 102 were developing, and 632 were impacted.

Status of maxillary third molars: The distribution of maxillary third molars (teeth 18 and 28) according to sex and age group is shown in Table 1. Absence was the most frequent finding for both teeth, followed by fully erupted and impacted third molars. Developing teeth were rare and mainly observed in younger age groups. Females showed a slightly higher number of absent and fully erupted maxillary third molars compared to males. Across age groups, impacted and developing maxillary third molars were more frequently observed in patients younger than 30 years, whereas absence predominated in patients older than 36 years.

Status of mandibular third molars: The distribution of mandibular third molars (teeth 38 and 48) is presented in Table 2. Compared to maxillary third molars, mandibular third molars showed a higher prevalence of impaction. Tooth 38 presented 200 impacted cases, while tooth 48 presented 197 impacted cases. As observed for maxillary teeth, developing M3M were almost exclusively found in younger patients. In individuals over 36 years of age, absence was the most common finding.

Overall prevalence of impacted third molars: Out of a total of 1811 third molars evaluated, 632 (34.9%) were impacted. Impacted third molars were more frequently located in the mandible (n = 397) than in the maxilla (n = 235).

Angulation of Impacted Third Molars: The angulation of impacted third molars according to Winter’s classification is summarized in Table 3. Vertical angulation was the most frequent pattern for all teeth, particularly for mandibular third molars (teeth 38 and 48). Mesioangular and distoangular impactions were also commonly observed, especially in the mandible.

Horizontal impactions were predominantly found in mandibular third molars, with 18 cases in tooth 38 and 20 cases in tooth 48.

Depth and Ramus Relationship of Impacted Third Molars: The distribution of impacted third molars according to the Pell and Gregory classification is shown in Table 4 and Table 5. For maxillary third molars (teeth 18 and 28), only depth can be assessed. Level C impaction was the most frequent depth classification.

Mandibular third molars were most classified as Level A, Class II and Level B, Class II. Level C impactions were also observed, particularly in Class II and Class III relationships, indicating deeper impactions and closer proximity to the mandibular ramus.

Pathological conditions associated with third molars: Radiographic signs of pathology associated with third molars are presented in Table 6. The most frequent findings were dental caries affecting third molars (n = 124) and second molars (n = 109), as well as periodontal pockets (n = 109).

Caries affecting third molars occurred predominantly in fully erupted teeth, whereas caries of the adjacent second molars were more frequently associated with impacted third molars (Table 7). Other findings included pericoronal radiolucency (n = 78), extrusion (n = 35), second molar root resorption (n = 26), and periapical lesions (n = 4). Supernumerary teeth were rarely observed (n = 3).

Radiographic Signs Suggestive of Inferior Alveolar Canal Proximity: Radiographic signs suggestive of proximity between the M3M and the IAC were first evaluated on OPGs, followed by CBCT evaluation to confirm contact (n = 205). The relationship between IAC contact and the presence of radiographic proximity signs to the IAC was statistically significant (*p* < 0.05).

The absence of contact was predominantly observed in cases without OPG radiographic signs of proximity (n = 70, 60%). In contrast, contact without cortical bone was more frequently associated with the radiographic signs of cortical interruption (n = 27, 36.5%) and darkening roots (n = 21, 28.4%). A comprehensive overview of the association between radiographic proximity signs and mandibular canal contact is presented in Table 8.

CBCT analysis was performed on 205 mandibular third molars (tooth 38: n = 100; tooth 48: n = 105).

Presence of Contact with the IAC and Cortical Condition: The relationship between the IAC contact and IAC spatial position in relation to the roots of mandibular impacted third molars is presented in Table 9.

Overall, 52.7% of M3Ms had no contact with the IAC, 12.2% were in contact with cortical bone present, and 36.1% demonstrated contact without the presence of cortical bone. Regarding the spatial position of the IAC relative to the tooth roots, the most frequently observed position was apical to the roots (73.3%), followed by buccal (13.0%) and lingual (11.4%) positions. When no contact was observed, the apical position was predominant.

Position of the IAC relative to the roots of the M3M and Minimal Distance: The minimal distance between mandibular third molar roots and the inferior alveolar canal ranged from 0.00 mm (direct contact) to 10.87 mm. Mean values were 2.01 ± 2.27 mm for tooth 38 and 1.58 ± 2.08 mm for tooth 48. No statistically significant differences were observed between sides (*p* = 0.156) (Table 10). This quantitative measure complements the positional analysis, highlighting that in many cases the nerve was in very close proximity to the tooth roots. Additionally, the associated effect size was small (Cohen’s d = 0.20), indicating the absence of a clinically meaningful difference between left and right mandibular third molars.

Lingual Plate Thickness: Mean lingual cortical plate thickness progressively decreased from the cervical to the apical region. At the cementoenamel junction, mean values were 2.42 ± 0.91 mm for tooth 38 and 2.38 ± 0.78 mm for tooth 48 (*p* = 0.704; Cohen’s d = 0.05). At the mid-root level, corresponding values were 2.05 ± 1.14 mm and 1.94 ± 0.98 mm (*p* = 0.469; Cohen’s d = 0.10), while at the apical level they were 1.92 ± 1.51 mm and 1.99 ± 1.41 mm (*p* = 0.705; Cohen’s d = 0.05) (Table 10). All effect sizes were negligible, confirming that observed differences between teeth 38 and 48 were neither statistically significant nor clinically relevant.

Overall, CBCT-derived measurements demonstrated anatomical symmetry between mandibular third molars 38 and 48, with negligible to small effect sizes across all continuous variables.

## 4. Discussion

This retrospective study provides a comprehensive epidemiological and three-dimensional radiographic characterization of impacted third molars in a Portuguese population, integrating a large OPG dataset with a CBCT subsample focused on surgically relevant anatomical parameters. This two-modality approach makes it possible not only to estimate prevalence and impaction configurations but also a clinically meaningful interpretation regarding anatomical risk factors associated with mandibular third molar surgery.

The comparison between left (38) and right (48) mandibular third molars was intentionally included to address a common clinical assumption that laterality may influence surgical difficulty and neurosensory risk. In routine clinical practice, asymmetrical panoramic radiographic signs are frequently observed and often interpreted as indicators of side-specific surgical risk. However, evidence supporting true three-dimensional anatomical asymmetry between mandibular sides remains limited. The present analysis, therefore, sought to determine whether such assumptions are substantiated when mandibular third molars are evaluated using CBCT-derived anatomical parameters.

The overall prevalence of impacted third molars, categorized at 34.9% in this study, is consistent with the range reported in recent systematic reviews and European cohort studies, which vary between 24% and 37% depending on parameters such as age distribution and diagnostic criteria [7,38,39]. Unsurprisingly, mandibular third molars were found to be impacted more frequently compared to maxillary third molars, reinforcing the role of spatial constraints, delayed eruption timing, and mandibular growth patterns in impaction etiology [40].

Vertical angulation showed the highest frequency of impaction in both jaws, especially in the mandible. Although some earlier epidemiological studies cited mesioangular impaction as the most common type of impaction [7], recent investigations—especially those involving older populations—have noted an increasing trend toward vertical impaction [41,42,43]. This shift may reflect arrested eruption following partial eruption or age-related changes in occlusal dynamics and alveolar bone remodeling. It must not be forgotten that dissimilarities in the values of different studies can be due to variations in anatomy or the criteria of selection and classification.

Assessing impaction angulation with Winter’s classification provides a limited view of surgical complexity, but combining it with the Pell and Gregory classification yields a more complete understanding of impaction patterns. The present sample showed a balanced distribution across depths. Both teeth 38 and 48 predominantly presented as Class IIA (35.4% and 34.7%, respectively), followed by Class IIB (29% and 30.6%, respectively).

The radiographic findings also highlight the clinical relevance of third molars as a source of both local and adjacent pathology. While caries affecting third molars were predominantly associated with fully erupted teeth, impacted third molars were more frequently linked to carious lesions in adjacent second molars, supporting their key role as a risk factor for distal caries development, in agreement with findings previously reported in the literature [44,45,46]. 

In the present study, cortical interruption and darkening of the roots were the panoramic signs most strongly associated with direct contact without the presence of cortical bone between the M3M and the IAC. These findings are consistent with other studies that also identified these features as the most reliable OPG indicators of IAC proximity [30,31,47,48]. These studies emphasize the diagnostic value of these predictors, while also underlining that the absence of OPG signs generally corresponds to the absence of contact on CBCT. In addition, Kim et al. described a periapical band-like radiolucent sign on OPG that was significantly associated, on CBCT evaluation, with close proximity of the mandibular third molar to the lingual cortical plate, supporting the concept that panoramic imaging may offer indirect screening cues for lingual cortical relationships in selected cases [29]. Recent studies indicate that panoramic signs provide only moderate diagnostic performance and that CBCT may improve three-dimensional risk stratification in selected high-risk cases, without supporting routine CBCT use in all patients [11,49,50,51].

A relevant methodological difference of the current study lies in the introduction of an additional classification differentiating cases of IAC contact with and without cortical integrity. Such subdivision provides a better surgical risk assessment and enhances prognosis analysis since it may considerably vary depending on the preservation of the cortical bone. As corroborated by other studies, these features represent reliable radiographic indicators of proximity to the IAC with meaningful implications for surgical risk assessment [28,51,52].

While OPGs are useful for screening, CBCT accurately reveals the buccolingual relationship and canal proximity, thus a major strength of the present study lies in the three-dimensional CBCT assessment of mandibular third molars in relation to the IAC. The anatomical relationship between the IAC and the roots of the M3Ms is a key factor of surgical risk, with the lingual position of the IAC and the loss of cortical integrity being major predictors of IAN injury [31,33,53,54,55].

The apical position of the IAC relative to the roots was the most frequently observed configuration, in agreement with previous CBCT-based investigations [32,53,56].

The mean minimal distance between the roots of M3M and IAC was 2.01 ± 2.27 for tooth 38 and 1.58 ± 2.08 for tooth 48. Shorter distances have consistently been associated with a greater risk of IAN injury during surgery, particularly when no cortical is present [32,53,56].

Lingual nerve injury is a well-recognized complication associated with M3M surgery. Taking into consideration the anatomical variability of the nerve’s path and the vulnerability to fracture of the lingual cortical plate, an accurate preoperative evaluation is critical to prevent the risk of nerve damage and sensitive alterations [30,57,58,59]. Within the context of the present study, a progressive decrease in lingual plate thickness was found from the cervical part to the apical area, as found in other studies [34,35]. Overall, mean lingual cortical thickness values were found to be comparable between sides at all anatomical levels considered, and perforation and/or thickness values below 1 mm were exceptionally recorded. The findings within the context of the current investigation confirm those found in other studies and suggest that the risk of lingual nerve damage is a multifactorial event that is not predicted by the thinness of the cortical plate alone, but is rather a conjunction of anatomical, technical, and surgeon-related factors [60,61,62,63].

Taken together, the combined interpretation of statistical significance and consistently negligible to small effect sizes indicates that mandibular third molar laterality is unlikely to represent a meaningful modifier of neurosensory risk. These findings challenge the common clinical assumption that left–right asymmetry influences surgical risk and reinforce that true three-dimensional anatomical relationships are largely symmetrical between mandibular sides. From a clinical perspective, individualized CBCT-based assessment of canal position, cortical integrity, and root proximity provides more relevant information for surgical planning than tooth laterality alone.

Some limitations of this study should be acknowledged. First, although the panoramic radiographic sample was large and appropriate for prevalence estimation, the CBCT analysis was restricted to a subset of cases obtained from a single clinical center, which may limit the external generalizability of the three-dimensional anatomical findings. In addition, the age distribution in the panoramic sample was skewed toward older patients, which may influence prevalence estimates and impact patterns. Second, CBCT scans were acquired using two different voxel sizes (0.20 mm and 0.40 mm). While both voxel dimensions are considered suitable for the assessment of IAC position and lingual cortical plate thickness, minor variability in linear measurements cannot be entirely excluded. Nevertheless, such variability is unlikely to be clinically relevant and is not expected to influence the main conclusions of anatomical symmetry and anatomy-based risk assessment. Finally, the retrospective design and the absence of postoperative neurosensory outcome data preclude direct correlation between radiographic findings and clinical complications, reinforcing that the present conclusions should be interpreted within an anatomical and diagnostic framework rather than as predictors of surgical outcomes.

Additionally, the age distribution of the OPG cohort was skewed towards older individuals, which may influence observed impaction patterns and prevalence estimates. However, this does not compromise the internal validity of the radiographic classification and three-dimensional anatomical assessments within the CBCT subsample.

## 5. Conclusions

In this Portuguese cohort, the prevalence of impacted third molars is consistent with recent epidemiological evidence, confirming a higher occurrence of impaction in the mandible and a predominance of vertical angulation. In the nested CBCT analysis, IAC position, contact status and cortical integrity provided clinically meaningful three-dimensional descriptors, while laterality showed no clinically relevant influence on key anatomical risk indicators. These findings support anatomy-driven, selective use of CBCT when OPG risk signs raise concern, and they should be interpreted as a radiographic risk assessment rather than evidence of postoperative neurosensory outcomes.

Three-dimensional CBCT analysis demonstrated that the apical position of the inferior alveolar canal relative to mandibular third molar roots is the most frequent anatomical configuration. No clinically relevant differences were identified between left and right mandibular third molars regarding root–canal distance or lingual cortical plate thickness, with negligible effect sizes supporting anatomical symmetry.

Overall, these findings support an anatomy-driven, patient-specific approach to surgical planning, reinforcing that mandibular third molar laterality alone should not influence clinical decision making in the absence of three-dimensional anatomical risk factors. When clinically justified, CBCT provides valuable information for accurate diagnosis and treatment planning, supporting safer, anatomy-informed surgical decision making.

## Figures and Tables

**Table 1:** Demographic characteristics considering the maxillary third molars status.

Tooth		18				28			
		Ab	FE	D	I	Ab	FE	D	I
Sex	Female	391 (62.3%)	154 (54%)	15 (50%)	59 (49.6%)	386 (62.1%)	160 (54.2%)	14 (50%)	59 (50.9%)
	Male	237 (37.7%)	131 (46%)	15 (50%)	60 (50.4%)	236 (37.9%)	135 (45.8%)	14 (50%)	57 (49.1%)
	Total	628 (100%)	285 (100%)	30 (100%)	119 (100%)	622 (100%)	295 (100%)	28 (100%)	116 (100%)
Age (years)	17–20	4 (0.6%)	5 (1.8%)	26 (86.7%)	19 (16%)	2 (0.3%)	8 (2.7%)	25 (89.3%)	19 (16.4%)
	21–25	11 (1.8%)	17 (6%)	2 (6.7%)	28 (23.5%)	16 (2.6%)	16 (5.4%)	1 (3.5%)	25 (21.6%)
	26–30	28 (4.5%)	24 (8.4%)	0 (0%)	11 (9.2%)	26 (4.2%)	28 (9.5%)	0 (0%)	9 (7.8%)
	31–35	48 (7.6%)	32 11.2%)	0 (0%)	14 (11.8%)	42 (6.8%)	36 (12.2%)	0 (0%)	16 (13.8%)
	≥36	537 (85.5%)	207 (72.6%)	2 (6.7%)	47 (39.5%)	536 (86.2%)	207 (70.2%)	2 (7.1%)	47 (40.5%)

Ab: Absent; FE: Fully erupted; D: Developing; I: Impacted.

**Table 2:** Demographic characteristics considering mandibular third molars status.

Tooth		48				38			
		Ab	FE	D	I	Ab	FE	D	I
Sex	Female	370 (61.9%)	136 (55.7%)	12 (52.2%)	101 (51.3%)	370 (62.9%)	137 (54.2%)	10 (47.6%)	102 (51%)
	Male	228 (38.1%)	108 (44.3%)	11 (47.8%)	96 (48.7%)	218 (37.1%)	116 (45.8%)	11 (52.4%)	98 (49%)
	Total	598 (100%)	244 (100%)	23 (100%)	197 (100%)	588 (100%)	253 (100%)	21 (100%)	200 (100%)
Age (years)	17–20	6 (1%)	2 (0.8%)	22 (95.7%)	24 (12.2%)	5 (0.9%)	2 (0.8%)	21 (100%)	26 (13%)
	21–25	13 (2.2%)	4 (1.6%)	1 (4.3%)	40 (20.3%)	15 (2.6%)	5 (2%)	0 (0%)	38 (19%)
	26–30	26 (4.3%)	12 (4.9%)	0 (0%)	25 (12.7%)	24 (4.1%)	9 (3.6%)	0 (0%)	30 (15%)
	31–35	48 (8%)	22 (9%)	0 (0%)	24 (12.2%)	45 (7.7%)	19 (7.5%)	0 (0%)	30 (15%)
	≥36	505 (84.4%)	204 (83.6%)	0 (0%)	84 (42.6%)	499 (84.9%)	218 (86.2%)	0 (0%)	76 (38%)

Ab: Absent; FE: Fully erupted; D: Developing; I: Impacted.

**Table 3:** Angulation of impacted third molars according to Winter’s Classification.

Tooth		18	28	38	48
Winter Classification	Vertical	54 (46.2%)	51 (44.3%)	82 (42.1%)	84 (42.9%)
	Mesioangular	26 (22.2%)	19 (16.5%)	51 (26.2%)	51 (26%)
	Distoangular	23 (19.7%)	28 (24.3%)	36 (18.5%)	34 (17.3%)
	Horizontal	3 (2.6%)	1 (0.9%)	18 (9.2%)	20 (10.2%)
	Other	11 (9.4%)	16 (13.9%)	8 (4.1%)	7 (3.6%)
Total		117 (100%)	115 (100%)	195 (100%)	196 (100%)

**Table 4:** Pell and Gregory Classification for maxillary impacted third molars.

Tooth		18	28
Pell and Gregory Classification	Level A	2 (1.7%)	2 (1.7%)
	Level B	26 (22.2%)	28 (24.3%)
	Level C	89 (76.1%)	85 (73.9%)
Total		117 (100%)	115 (100%)

**Table 5:** Pell and Gregory Classification for mandibular impacted third molars.

Tooth		48	38
Pell and Gregory Classification	Level A, Class I	3 (1.5%)	5 (2.6%)
	Level A, Class II	68 (34.7%)	69 (35.4%)
	Level A, Class III	1 (0.5%)	3 (1.5%)
	Level B, Class I	8 (4.1%)	8 (4.1%)
	Level B, Class II	60 (30.6%)	57 (29%)
	Level B, Class III	12 (6.1%)	12 (6.2%)
	Level C, Class I	5 (2.6%)	4 (2.1%)
	Level C, Class II	25 (12.8%)	21 (10.8%)
	Level C, Class III	14 (7.1%)	16 (8.2%)
Total		196 (100%)	195 (100%)

**Table 6:** Radiographic signs of pathology associated with third molars.

Tooth		18	28	38	48	Total
Pathology	Second Molar Carie	21 (19.3%)	24 (22%)	36 (33%)	38 (34.9%)	109 (100%)
	Third Molar Carie	35 (28.2%)	33 (26.6%)	32 (25.8%)	24 (19.4%	124 (100%)
	Root resorption of second molars	8 (30.8%)	6 (23.1%)	7 (26.9%)	5 (19.2%)	26 (100%)
	Pericoronal radiolucency	6 (7.7%)	6 (7.7%)	31 (39.7%)	35 (44.9%)	78 (100%)
	Supernumerary	2 (66.7%)	1 (33.3%)	-	-	3 (100%)
	Extrusion	17 (48.6%)	11 (31.4%)	3 (8.6%)	4 (11.4%)	35 (100%)
	Periodontal Pocket	36 (33%)	28 (25.7%)	21 (19.2%)	24 (22%)	109 (100%)
	Periapical Lesion	-	-	3 (75%)	1 (25%)	4 (100%)

**Table 7:** Caries affecting second and third molars according to eruption status.

	Second Molar Carie	Third Molar Carie	Total
Fully Erupted	55 (5.1%)	98 (9.1%)	**1077**
Impacted	54 (8.5%)	26 (4.1%)	**632**

**Table 8:** Association between radiographic signs suggestive of IAC proximity on OPG and IAC contact confirmed on CBCT (N = 205).

OPG Proximity Signs	IAC Contact			Total
	No	Yes with Cortical	Yes Without Cortical	
None	70 (66%)	5 (20%)	12 (16.2%)	87 (42.4%)
Narrowing canal	5 (4.7%)	4 (16%)	6 (8.1%)	15 (7.3%)
Narrowing roots	2 (1.9%)	1 (4%)	3 (4.1%)	6 (2.9%)
Darkening roots	13 (12.3%)	8 (32%)	21 (28.4%)	42 (20.5%)
Canal deviation	1 (0.9%)	1 (4%)	0 (0%)	2 (1%)
Cortical interruption	14 (13.2%)	5 (20%)	27 (36.5%)	46 (22.4%)
Root deflection	1 (0.9%)	1 (4%)	5 (6.8%)	7 (3.4%)
Total	106 (100%)	25 (100%)	74 (100%)	205 (100%)

IAC: inferior alveolar canal; OPG: orthopantomography.

**Table 9:** Relationship between the IAC contact and the IAC position assessed on CBCT (n = 205).

48		IAC Position				Total
		Apical	Buccal	Lingual	Interradicular	
IAC Contact	Yes with cortical	10 (9.5%)	2 (1.9%)	3 (2.9%)	-	15
	Yes without cortical	27 (25.7%)	4 (3.8%)	8 (7.6%)	1 (1%)	40
	No	40 (38.1%)	9 (8.6%)	1 (1%)	-	50
Total		77	15	12	1	105
**38**		**IAC Position**				**Total**
		**Apical**	**Buccal**	**Lingual**	**Interradicular**	
IAC Contact	Yes with cortical	9 (9%)	1 (1%)	-	-	10
	Yes without cortical	18 (18%)	2 (2%)	12 (12%)	2 (2%)	34
	No	51 (51%)	3 (3%)	2 (2%)	-	56
Total		78	6	14	2	100

IAC: inferior alveolar canal.

**Table 10:** Summarizes the descriptive statistics for continuous CBCT variables. No statistically significant differences were observed between mandibular third molars 38 and 48 (*p* > 0.05). All effect sizes were negligible to small, indicating no clinically meaningful anatomical asymmetry.

Variable	Tooth 38 (Mean ± SD)	Tooth 48 (Mean ± SD)	*p*-Value	Cohen’s d	Effect Size
Minimal distance root–IAC (mm)	2.01 ± 2.27	1.58 ± 2.08	0.156	0.20	Small
Lingual cortical thickness at CEJ (mm)	2.42 ± 0.91	2.38 ± 0.78	0.704	0.05	Negligible
Lingual cortical thickness at mid-root (mm)	2.05 ± 1.14	1.94 ± 0.98	0.469	0.10	Negligible
Lingual cortical thickness at root apex (mm)	1.92 ± 1.51	1.99 ± 1.41	0.705	0.05	Negligible

IAC: inferior alveolar canal; CEJ: cement-enamel junction; SD: standard deviation.

## Data Availability

The original contributions presented in this study are included in the article. Further inquiries can be directed to the corresponding authors.

## Abbreviations

The following abbreviations are used in this manuscript:

CBCT	Cone-beam computed tomography
IAC	Inferior alveolar canal
IAN	Inferior alveolar nerve
LN	Lingual Nerve
M3M	Mandibular third molar
Mx3M	Maxillary third molar
OPG	Orthopantomography
